# Mortality Prediction of Patients With Cardiovascular Disease Using Medical Claims Data Under Artificial Intelligence Architectures: Validation Study

**DOI:** 10.2196/25000

**Published:** 2021-04-01

**Authors:** Linh Tran, Lianhua Chi, Alessio Bonti, Mohamed Abdelrazek, Yi-Ping Phoebe Chen

**Affiliations:** 1 School of Info Technology Deakin University Burwood Australia; 2 Department of Computer Science and Information Technology La Trobe University Bundoora Australia

**Keywords:** mortality, cardiovascular, medical claims data, imbalanced data, machine learning, deep learning

## Abstract

**Background:**

Cardiovascular disease (CVD) is the greatest health problem in Australia, which kills more people than any other disease and incurs enormous costs for the health care system. In this study, we present a benchmark comparison of various artificial intelligence (AI) architectures for predicting the mortality rate of patients with CVD using structured medical claims data. Compared with other research in the clinical literature, our models are more efficient because we use a smaller number of features, and this study could help health professionals accurately choose AI models to predict mortality among patients with CVD using only claims data before a clinic visit.

**Objective:**

This study aims to support health clinicians in accurately predicting mortality among patients with CVD using only claims data before a clinic visit.

**Methods:**

The data set was obtained from the Medicare Benefits Scheme and Pharmaceutical Benefits Scheme service information in the period between 2004 and 2014, released by the Department of Health Australia in 2016. It included 346,201 records, corresponding to 346,201 patients. A total of five AI algorithms, including four classical machine learning algorithms (logistic regression [LR], random forest [RF], extra trees [ET], and gradient boosting trees [GBT]) and a deep learning algorithm, which is a densely connected neural network (DNN), were developed and compared in this study. In addition, because of the minority of *deceased* patients in the data set, a separate experiment using the Synthetic Minority Oversampling Technique (SMOTE) was conducted to enrich the data.

**Results:**

Regarding model performance, in terms of discrimination, GBT and RF were the models with the highest area under the receiver operating characteristic curve (97.8% and 97.7%, respectively), followed by ET (96.8%) and LR (96.4%), whereas DNN was the least discriminative (95.3%). In terms of reliability, LR predictions were the least calibrated compared with the other four algorithms. In this study, despite increasing the training time, SMOTE was proven to further improve the model performance of LR, whereas other algorithms, especially GBT and DNN, worked well with class imbalanced data.

**Conclusions:**

Compared with other research in the clinical literature involving AI models using claims data to predict patient health outcomes, our models are more efficient because we use a smaller number of features but still achieve high performance. This study could help health professionals accurately choose AI models to predict mortality among patients with CVD using only claims data before a clinic visit.

## Introduction

### Background

In Australia, cardiovascular disease (CVD) is the most concerning health problem, killing more people than any other disease and placing heavy burdens on the health care system because of enormous costs and on individuals and the community owing to resulting disabilities. CVD was the leading cause of death among Australians in 1997, accounting for 52,641 deaths, 41% of all deaths [1]. An estimated 1.2 million (5.6%) Australian adults aged 18 years and more had one or more conditions related to heart or vascular disease, including stroke, in 2017-2018, based on self-reported data from the Australian Bureau of Statistics 2017-2018 National Health Survey. The prevalence of CVD by age group and sex, in 2017-2018, is shown in Table 1.

**Table 1:** Prevalence of cardiovascular disease by age group and sex, 2017-2018.

Age group (years)	Men, n^a^	Women, n^a^	Total, n^a^	Men, % (95% CI)^b^	Women, % (95% CI)^b^	Total, % (95% CI)^b^
18-44	31,400	56,600	88,000	0.7 (0.3-1.1)	1.2 (0.7-1.8)	1.0 (0.7-1.3)
45-54	50,600	42,300	92,900	3.3 (2.4-4.2)	2.6 (1.7-3.5)	3.0 (2.4-3.6)
55-64	136,700	114,700	251,500	10.0 (7.6-12.4)	7.9 (6.0-9.9)	8.9 (7.4-10.5)
65-74	208,900	135,600	344,500	19.8 (17.2-22.4)	12.2 (10.0-14.4)	15.9 (14.3-17.5)
75+	213,200	160,100	373,300	32.1 (27.1-37.0)	20.3 (17.5-23.1)	25.7 (23.1-28.2)
Persons (number/age-standardized rate^c^)	640,800	509,300	1,150,200	6.5 (5.9-7.0)	4.8 (4.3-5.3)	5.6 (5.2-5.9)

^a^Due to rounding, discrepancies may occur between sums of the component items and totals.

^b^CI is a statistical term describing a range (interval) of values within which we can be “confident” that the true value lies, usually because it has a 95% or higher chance of doing so.

^c^Age-standardized to the 2001 Australian Standard Population (Source: AIHW analysis of ABS 2019).

The major risk factors for CVD are tobacco smoking, high blood pressure, high blood cholesterol, overweight, insufficient physical activity, high alcohol use, and type 2 diabetes [1]. CVD treatments are usually prescribed in combination with other drugs such as antidiabetics, antihypertensives, lipid-lowering drugs, anticoagulants, and antiplatelet agents [2]. Medication use is an important management factor for patients diagnosed with heart disease besides eating a healthy diet and maintaining fitness with regular physical activity. Medications are used to minimize symptoms, reduce the risk of exacerbation, and improve the quality of life.

Many methods have been developed to predict the mortality rate of patients with CVD by using many algorithms and predictor variables. There are 3 main methods for forecasting mortality: explanation, expectation, and extrapolation [3]. Of these, the most common basis of forecasting mortality is extrapolation, which assumes that the future state is highly correlated to the past. In the clinical literature, historical electronic health records (EHRs) are widely used to develop artificial intelligence (AI) models that can predict the health outcomes of patients. Information commonly extracted from EHR as input for AI models includes patient demographics, health indices, medical conditions, biomedical images, or clinical notes, whereas structured medical claims data are rarely used. Although medical claims data inadequately inform patient health conditions, this source of information is crucial in reflecting patient health care access frequency and level of participation in disease prevention or treatment, which has a great impact on patient health outcomes.

In this study, we present a benchmark comparison of the performance of different AI architectures: 4 classical machine learning (ML) algorithms (logistic regression [LR], random forest [RF], extra trees [ET], and gradient boosting trees [GBT]) and a deep learning algorithm, which is a densely connected neural network (DNN) that uses medical scheduling and pharmaceutical dispensing information from historical claims data to predict the mortality rate of patients with CVD. Compared with other research in the clinical literature involving AI models using claims data to predict patient health outcomes, our models are more efficient because we use a smaller number of features but still achieve high performance. Furthermore, we also propose Synthetic Minority Oversampling Technique (SMOTE), a technique to enrich training data and handle class imbalance, as a tool to improve the performance of the developed AI models.

### Related Work

Recent trends involve using AI models to learn patterns from large data sets to predict mortality with higher accuracy [4]. The American College of Cardiology Foundation’s National Cardiovascular Data Entry conducted a study that used statistical analysis to predict the rate of risk in percutaneous coronary intervention. The study results show that ML models perform better in terms of accuracy than classical statistical models [5]. One study showed that ML models such as RF, decision tree, and LR perform exceptionally well owing to today’s computational power, which allows them to process data from the electrical health records [6] of patients. ML models deployed on routine clinical data performed better than standard cardiovascular risk assessment models and had great merits in terms of preventive treatment and avoidance of mistreatment for CVD according to a study conducted on a large sample of patients in the United Kingdom [7]. Moreover, using neural networks for predictive analysis of illnesses was shown to be fruitful as early as in 2005 [8]. Wang et al [9] predicted the mortality rate because of heart failure by deploying a convolutional, layered neural network that inculcated feature rearrangement to select the best features. Another study has shown that deep neural networks perform better than traditional ML models with respect to accuracy and available sample size [10].

Many factors have been considered to predict the health outcomes of patients with heart disease. Some techniques used to extract learning features include automated imaging interpretation [11,12], natural language processing or text mining [13,14], and EHR extraction [15-18]. Imaging interpretation has been carried out by using deep neural networks [12] with promising results. Natural language processing of clinical notes has been shown to be able to correctly identify risks of CVD patients [13], whereas systematic application of text mining to the EHR has had variable success in the detection of cardiovascular phenotype [14]. It has been proven that applying ML helps identify clinically relevant patterns in the data [19]. Feature extraction from EHR allows the use of many factors, such as patient demographics, characteristics, and health conditions, including cardiovascular health (CVH) indices [20] or percutaneous coronary intervention indices [16,17] in predicting mortality risks.

On the basis of these studies, the mortality rate of patients in the cardiology cohort has been accurately predicted using a variety of algorithms, methods, and predictor features. However, there has been little focus on using medical claims to predict the health outcomes of patients with CVD. This information reflects patient medication usage, health care access frequency, and level of participation in disease prevention or treatment, which have a great impact on the determination of patient health outcomes [21]. Hence, to close this literature gap, in this study, mortality will be predicted based on patient medical schedule information and pharmaceutical dispensing history acquired from medical claims.

The Pharmaceutical Benefits Scheme (PBS) and Medicare Benefits Schedule (MBS) claims data collected by the Department of Human Services and held by the Department of Health have great potential to provide further insight into the medical scheduling and pharmaceutical dispensing history of patients with CVD. This study uses the PBS and MBS claims data in the period between 2004 and 2014 to investigate the mortality rate of patients with heart disease conditions in Australia and to build and compare 5 AI models to predict the mortality risk of a patient under these conditions. We built prediction models based on the patient’s age, gender, relevant medication prescriptions, medical schedule information, and pharmaceutical dispensing history obtained from the data set. We then assessed and compared the performance of each model and suggested recommendations for future work.

### Objectives

The primary aim of this research is to support health clinicians to accurately predict mortality among patients with CVD using only claims data before a clinic visit. Compared with other research in the clinical literature involving AI models using claims data to predict patient health outcomes, our models are more efficient because we use a smaller number of features but still achieve high performance. This study has applications in supporting health clinicians to accurately predict mortality among patients with CVD using only claims data before a clinic visit.

## Methods

### AI Architectures

In this study, 4 classical ML algorithm architectures, LR, RF, ET, and GBT, along with a deep learning algorithm called DNN were used to develop mortality prediction models. The MBS and PBS data sets are well structured and very informative and allows simple algorithms to learn better. Because our study deals with a probabilistic prediction problem, we put more emphasis on the discrimination and calibration of the model performance. Through initial experiments we found that LR, RF, ET, and GBT are classical ML algorithms that produce the best performance in terms of these two criteria. On the other hand, we were curious about how a state-of-the-art deep learning algorithm might perform on the data set. We developed the simplest neural network, a DNN, for further comparison and insights. We chose not to develop more complex deep learning architectures such as RNN or CNN because these algorithms are not necessary for such structured data sets to perform well. In this section, these experimental algorithms are described and their architectures proposed.

### Logistic Regression

LR is a supervised ML algorithm. It is a powerful and well-established method for binary classification problems [22]. LR is extended based on linear regression and can be used to calculate the probability of an event that has 2 possible outcomes by assigning weights to a number of predictor variables (features). Given a set of independent variables

x_1_,x_2_,x_3_,…,x_n_** (1)**

and a dependent variable *y*, which takes values between 0 and 1, first, LR is designed to find a set of weights

b_1_,b_2_,b_3_,...,b_n_** (2)**

for each of the independent variables so that the following linear equation outputs a logit score:

logit = b_0_ + b_1_x_1_ + b_2_x_2_ + b_3_x_3_ + ... + b_n_x_n_** (3)**

From this logit score, probability y is then derived by the following formula:


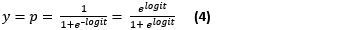


To use the LR as a binary classifier, a threshold must be assigned to differentiate between 2 classes. Normally, LR will classify an input instance with P>.50 as a positive class; otherwise, it is classified as a negative class. Depending on the problem, 0 and 1 can be translated into different meanings.

### Random Forest

**Figure 1:**
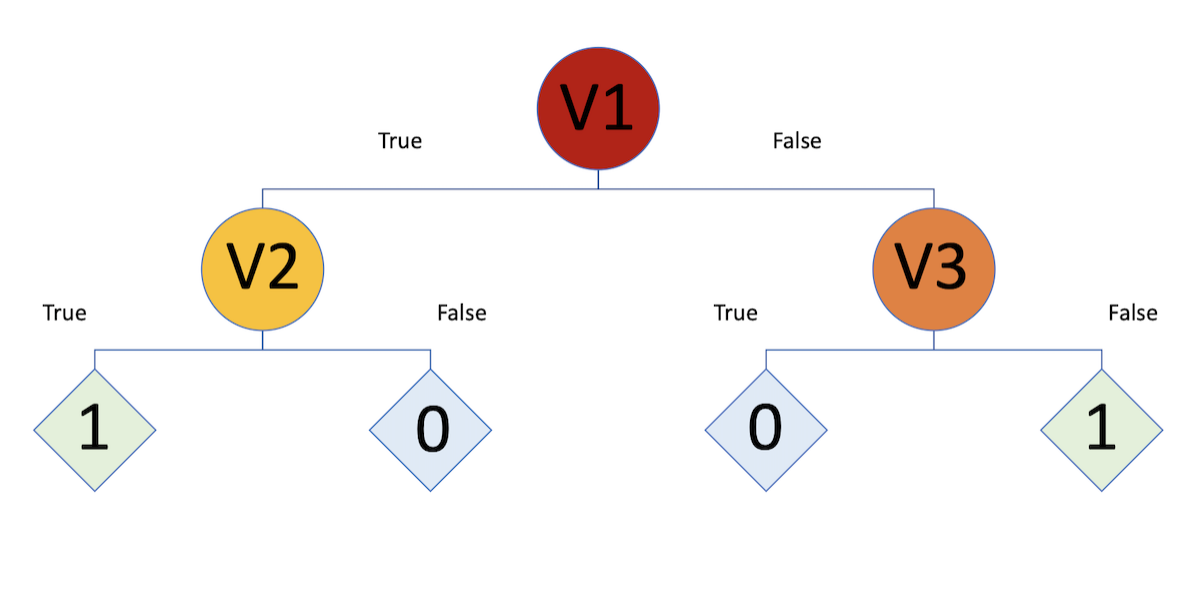
Decision tree.

Before describing the RF algorithm, it is important to understand the concept of the decision tree algorithm [23]. DT is one of the simplest and earliest ML algorithms. It structures the decision logic into a tree-like model. The nodes in a DT tree are partitioned into different levels, where the uppermost node is called the root node, whereas other nodes that have at least one child represent tests on input variables/features [24]. Depending on some criterion of the test, higher nodes are split into lower nodes repeatedly toward the leaf nodes [25], which have no child at all and correspond to the decision outcomes. An illustration of a simple DT is shown in Figure 1. According to Figure 1, the 3 circles -Sex, Age, and *A10*- are tested on the corresponding input variables, whereas the rhombuses at the end are the classification outcomes (*deceased* or *alive*).

**Figure 2:**
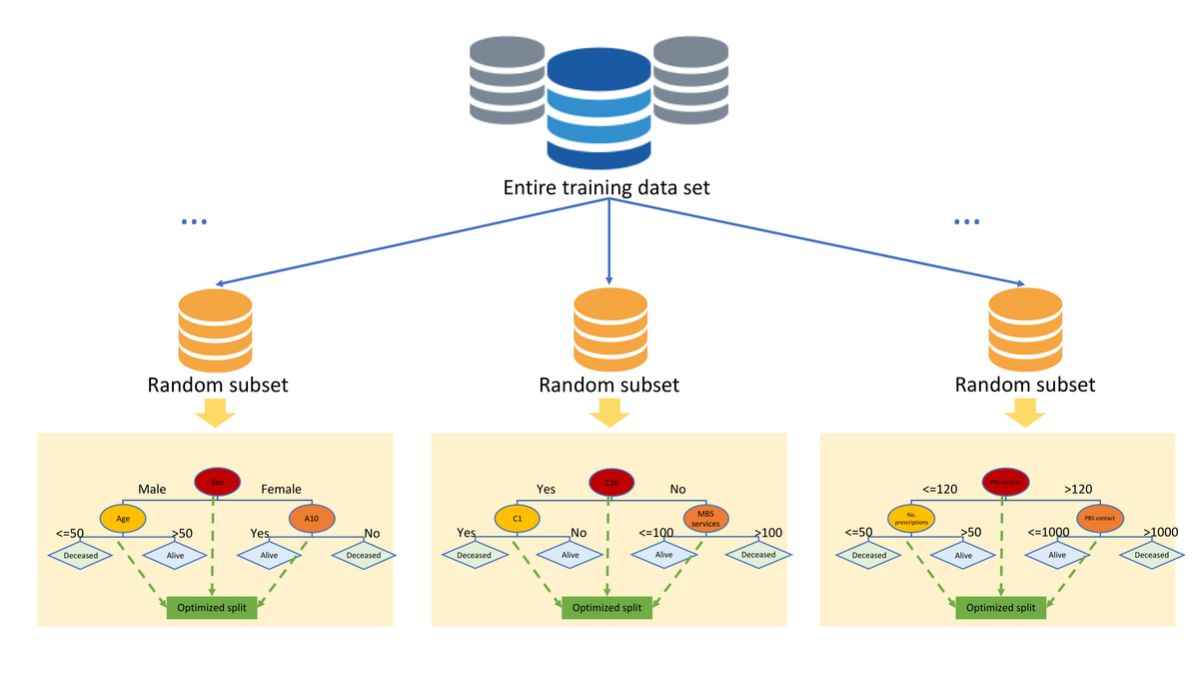
Random forests.

An RF is an ensemble classifier consisting of many DTs similar to a forest with many trees [26]. Different DTs in an RF are trained using different parts of the training data set and tested on different subsets of input variables. To classify a new instance, the input vector of the instance is pushed through each DT in the forest. Each DT makes decisions on a different part of the input vector and provides a classification outcome. The forest then makes a final prediction by majority vote in classification problems and by arithmetic average in regression problems. Because the RF algorithm aggregates outcomes from many different DTs to make a decision, the result has a smaller variance compared with the consideration of a single DT for the same data set. In addition, similar to other tree-based ensembles, variables for each tree in RF are randomized, whereas node-splitting cut points are locally optimized according to the criterion [26]. Figure 2 illustrates the RF algorithm. As shown in Figure 2, the training data set is randomly split into the desired number of trees in the forest, and each random subsample is then used to train a decision tree that is tested on a randomly selected subset of input variables.

### Extra Trees

**Figure 3:**
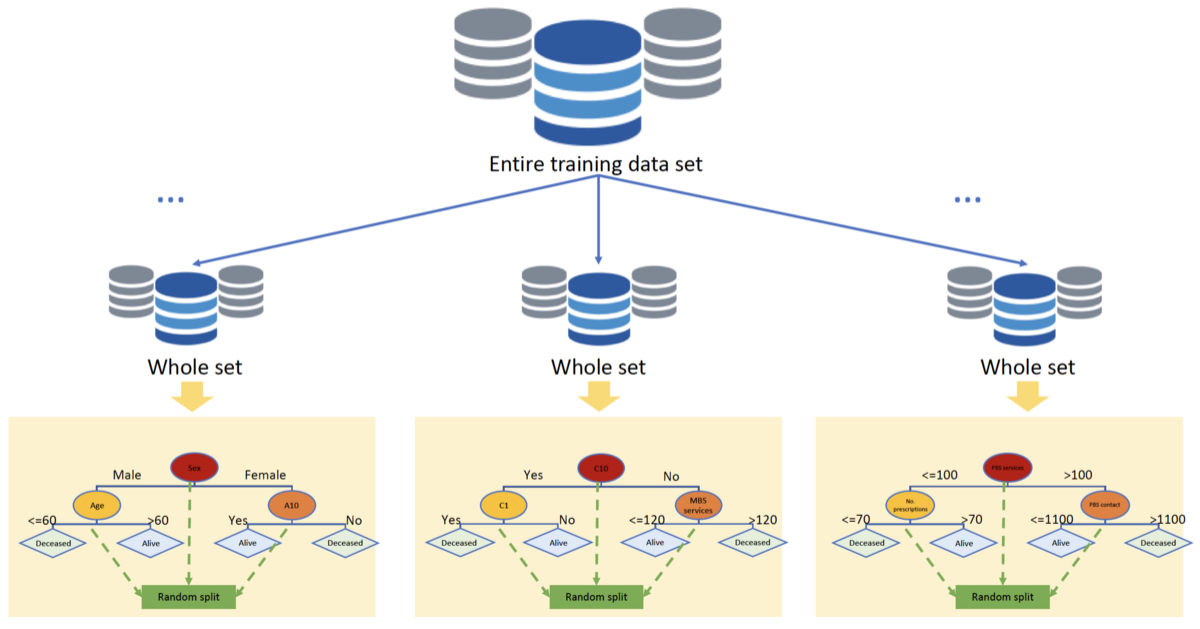
Extra trees.

The extremely randomized trees or the ET algorithm is also an ensemble classifier consisting of many single DTs similar to RF. The ET method also uses a random subset of features to train each base estimator [27]. However, the two main differences between RF and other tree-based ensemble methods are that RF splits nodes by choosing cut points fully at random (or random selection of threshold), and RF uses the whole learning sample to grow each tree in the ensemble rather than a subset of training data [28]. The final prediction produced is the aggregate of the predictions of all trained trees, yielded by the majority vote or arithmetic average in classification problems or regression problems, respectively. In terms of bias variance, ET is able to reduce the variance more effectively than the weaker randomization schemes used by other ensemble methods. On the other hand, a full training sample rather than bootstrap batches is used to train each base estimator in an attempt to minimize bias [28]. A simple illustration of the ET model is shown in Figure 3.

### Gradient Boosting Trees

**Figure 4:**
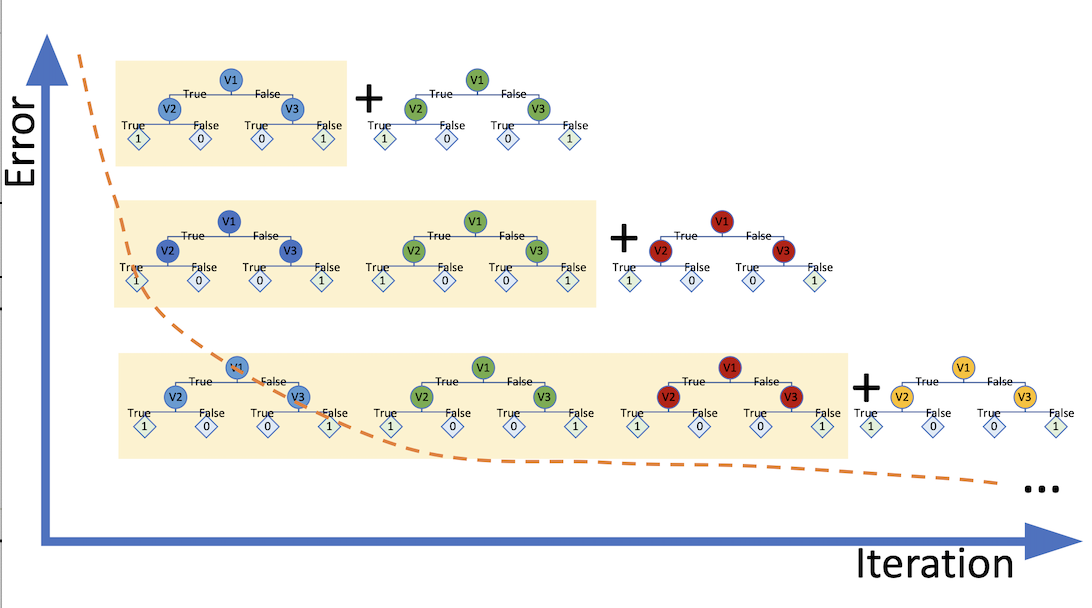
Gradient boosting trees workflow.

GBT is another popular ML algorithm that uses a tree-based ensemble method, and was first proposed by Friedman [29]. This approach trains learners (decision trees) by minimizing the loss function, which is computed using the gradient descent method [30]. To train a GBT, the algorithm first selects a very simple decision tree from the learning sample with equal weights. On the basis of the results of this weak learner, it tries to create a new learner who assigns higher weights to nodes that are more difficult to split and lower weights to those that are easier to split [30]. By doing this, the new learner is able to minimize the errors of the previous learner. As this process continues, the loss function is optimized [29], making each new model have a better goodness of fit with the observation data. Figure 4 illustrates the mechanism of the GBT algorithm.

### Densely Connected Neural Network

**Figure 5:**
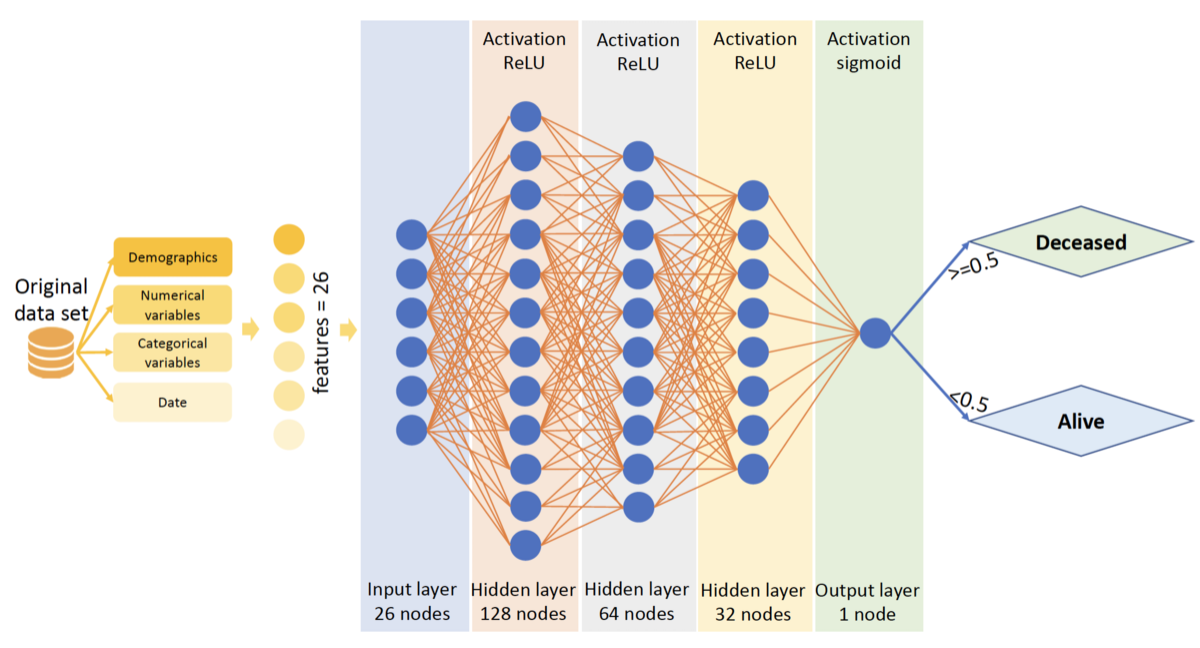
Artificial neural network architecture. ReLU: Rectified Linear Unit.

An artificial neural network (ANN) [3] is a deep learning architecture that replicates the neuron system inside the human brain. McCulloch and Pitts [31] first proposed ANN, and the concept was later popularized by the research work of Rumelhart et al [32]. In the human brain, neurons are linked together by numerous axon connections [33] and are responsible for adapting, processing, and storing information toward (inputs) and away (outputs) from the brain. Likewise, an ANN has hundreds or even thousands of artificial neurons called processing units, which are interconnected by nodes. In the ANN architecture, nodes are grouped into layers, depending on the activation they implement on the data. In the ANN, the output of one node is the input to another node. Subsequently, the input node after receiving information from the previous output node, based on an internal weighting system, attempts to produce the next output. Through repeated training, the weight system can amplify or weaken the level of communication between nodes. After mature training, which optimizes the weight system, a trained ANN can predict the test data. Because ANNs can be constructed by many layers and neurons, this method is considered a deep learning algorithm. Many types of ANNs are currently used in the literature, including feedforward neural networks, recurrent neural networks, convolutional neural networks, and modular neural networks. In this study, because our input data are well structured, allowing a neural network to learn effectively, we present the simplest form of ANN, which is a DNN. Figure 5 shows an illustration of the proposed DNN with 3 hidden layers.

## Results

### Benchmark Data

On August 1, 2016, the Department of Health released approximately 1 billion lines of anonymous historical health data relating to approximately 3 million Australians on data.gov.au. The information released includes details on medical services provided to Australians by health professionals, along with details of subsidized information. Claims data for a random 10% sample of Australians are made available for research institutions, health professionals, and universities. The data release includes historical medicare data (from 1984) and PBS data (from 2003) up to 2014. The release comprises 2 files corresponding to the 2 types of service information (MBS and PBS) and a separate patient demographic file. The data set used in this study was obtained from the MBS and PBS service information and patient demographic data by patient IDs. It originally included 346,201 records corresponding to 346,201 patients; however, 19 patients who had inadequate information were removed. Following this exclusion, the final data set comprised a total of 346,182 patients.

The data set included four classes of variables (ie, features):

1. Demographic variables: year of birth, sex, and age (calculated until January 1, 2015).
2. Numerical variables: A total of 13 continuous measurements are presented in the data set, including the number of MBS records, number of states, total amount of medical fees charged, total amount of medicare schedule fees, total amount of medical rebates paid, total number of MBS services, total duration of patients accessing medical services, number of PBS records, number of patient’s PBS codes, total amount of medication cost paid by the government, total amount of medication cost self-paid, total number of prescriptions, and total duration of patients accessing PBS services.
3. Categorical variables: These are 3 relevant medications classified by the Anatomical Therapeutic Chemical code and patient state. The medications presented are drugs used in diabetes (code: A10), drugs used for the cardiovascular system and hypertension (code: C0), and lipid-modifying agents or drugs used for patients with high cholesterol (code: C10).
4. Date variables: The 4 date variables include the date of the first medical schedule, date of the last medical schedule, date of the first PBS claim, and date of the last PBS claim.

Among these variables, except for the year of birth, age, and numerical variables that were kept constant, other variables were transformed as follows: sex and medication variables were mapped into binary values, whereas patient state was converted into 6 binary variables corresponding to 6 states. The year of birth, date of first medical schedule, and date of first PBS claim were used to calculate the age at which the patient had the first medical schedule and the first PBS claim, respectively, and then removed. Regarding the prediction target variable, because PBS and MBS claim data on their own do not include information about patients’ health outcomes, the labels must be inferred. Between the date of the last medical schedule and date of the last PBS claim, the latter was used to calculate the duration of patients discontinuing PBS and MBS services until January 1, 2015. Following this calculation, any patient who discontinued PBS and MBS for more than 180 days (6 months) was labeled *deceased*, otherwise *alive*. After preprocessing, the data set had 26 features and 1 label that used for model development.

In terms of feature scaling, each feature value was standardized to center around its mean with a unit standard deviation. This means that the mean of the attribute becomes zero and the resultant distribution has a unit standard deviation [34]. This step allows the algorithm to learn effectively as it eliminates sensitivity to multiple features spanning varying degrees of magnitude, range, and units.

In terms of class distributions, there are only 93,164 patients out of the total number of 346,182 classified into the *deceased* group, whereas the rest are *alive* patients. This reflects a highly imbalanced class distribution, which might affect the learning performance of the infrequent class [35] because of the lack of samples. To address this issue, a separate experiment using SMOTE was conducted as a trial to enrich the training set.

### Evaluation Metrics

Descriptive statistics were used to learn the characteristics of the study population, stratified by health outcome status (ie, alive or deceased). Models were derived from the training set and then assessed on the testing set by calculating the traditional accuracy, precision, and recall scores with the addition of brier loss. In addition, reporting discrimination and calibration is important for assessing a prediction model [36]. The area under the receiver operating characteristic curve (AUROC) score and the plotting reliability diagram (calibration curves) were also calculated to assess the performance of the AI models.

- Brier loss from scikit-learn measures the accuracy of probabilistic predictions by calculating the mean squared difference between the predicted probability assigned to the possible classes and the actual classes. It is composed of refinement loss and calibration loss so that the lower the Brier score is for a set of predictions, the better the predictions are calibrated or the better the model is.
- The AUROC score is used to measure the probability that the model ranks a random deceased patient higher than a random alive patient in terms of mortality rate. A higher AUROC score means that the model has a better ability to discriminate between deceased and alive populations.
- Calibration curve, a reliability diagram, is a line plot of the relative frequency of what was observed versus the predicted probability frequency. The closer the points appear along the main diagonal from the bottom left to the top right, the better calibrated a forecast or more reliable a model [37].

### Hyperparameters

To develop the models, the study population was stratified into a training set, in which the mortality risk algorithms were derived, and a testing set, in which the algorithms were applied and tested. The training set consisted of 90.00% (311,564/346,182) of the study data set, and the testing set consisted of the remaining 10.00% (34,618/346,182). The training and testing sets were split at the patient level and in a stratifying manner according to class ratio so that patients did not appear in both the training and testing sets and the ratio of patient labels (*deceased* or *alive*) in both sets were equivalent to that of the study population. After stratified assignment, the hyperparameters were determined by using a grid search of 5-fold cross-validation to determine the values that led to the best accuracy. After the grid search, each algorithm was refitted to the training set with its best hyperparameters to derive the final models. Table 2 presents the parameter search space of the 4 algorithms and the grid results.

**Table 2:** Hyperparameters for grid search.

Algorithms and parameter name			Search space		Optimal
**Logistic regression**					
	Penalty C tol solver multi_class	(‘l1’, ‘l2’, ‘none’) (0.01, 0.1, 1.0) (0.0001, 0.001, 0.01) (‘lbfgs’, ‘liblinear’, ‘sag’, ‘saga’) (‘auto’, ‘ovr’, ‘multinomial’)		l2 1.0 0.0001 lbfgs auto	
**Random forest**					
	n_estimators max_depth max_features min_samples_splitmin_samples_leaf	(5, 10, 50, 100, 150) (1, 2, 3, 5, None) (’auto’, ’sqrt’) (2, 5, 10) (1, 2, 4)		100 None auto 2 1	
**Extra trees**					
	n_estimators max_depth max_features min_samples_splitmin_samples_leaf	(5, 10, 50, 100, 150) (1, 2, 3, 5, None) (’auto’, ’sqrt’) (2, 5, 10) (1, 2, 4)		100 None auto 2 1	
**Gradient boosting trees**					
	Loss n_estimators max_depth learning_rate criterion	(‘deviance’, ‘exponential’) (5, 10, 50, 100, 150) (1, 2, 3, 5) (0.001, 0.01, 0.1) (’friedman_mse’, ’mse’, ’mae’)		deviance 100 3 0.1 friedman_mse	

After the grid search, it was found that LR with L2 regularization, which is also known as Ridge Regression [38], produces the most accurate predictions in cross-validation, with the C value and tolerance rate of 1.0 and 0.0001, respectively. This can be explained by the fact that our data set had a small number of features, making L1 regularization, which is Lasso Regression and works well for feature selection in a data set with high dimensionality [39], less favorable. Next, both RF and ET achieved optimal accuracy after grid search with the max_depth *None* scheme. According to the scikit-learn team, in this scheme nodes are expanded until all leaves are pure or until all leaves contain less than min_samples_split samples, which is optimized at 2 in both cases. Besides, the number of trees grown in both algorithms is the same, 100 (n_estimators). Last, errors in GBT are minimized using the deviance loss function; there are also 100 trees built with the maximum number of nodes equal to 3.

To develop the DNN model, the study population was stratified into training and testing sets with ratios of 90% and 10%, respectively. The training set was then broken down into training and validation sets with the same ratio. The purpose of the validation set was to provide an unbiased evaluation of the model while tuning the weights of the model [40]. The input layer had 26 units corresponding to the number of features, whereas the output layer had one unit. At the last step, sigmoid was used as the activation function to return the sigmoid values of the final output. The architecture of the DNN used is composed of 3 fully connected hidden layers. The number of neurons in each hidden layer are 128, 64 and 32, respectively, and the rectified linear unit is used as the activation function. During the training process, the parameters of the DNN are initialized using uniform initialization [41]. For each batch of training data, parameters of the DNN were modified gradually to decrease the cross-entropy of the loss function. A callback was set to stop the training process after 10 epochs when the model reached the highest value of AUROC.

After the training process, all models were evaluated using the holdout (10%) testing set. The final results were compared and used to make recommendations.

### Model Performance

In our experiments, we trained the models using the original learning sample and then applied SMOTE to further improve their performance.

#### Performance Without SMOTE

The details of the model performance without SMOTE are presented in Table 3. After adjusting for multiple comparisons, there was no significant difference in accuracy among RF (98.5%), GBT (98.4%), LR (97.8%), ET (97.9%), and DNN (97.1%). In terms of discrimination, GBT and RF achieved the highest AUROC (97.8% and 97.7%, respectively), followed by LR and ET (96.4% and 96.8%, respectively), whereas DNN was the least discriminative (95.3%). In terms of brier loss, GBT and RF produced the smallest difference between the probability assigned to the predicted classes and the probability of the actual class (both 0.012), whereas DNN predictions showed the largest difference (0.024).

**Table 3:** Performance metrics of machine learning models without the Synthetic Minority Oversampling Technique.

Algorithms	Accuracy	Area under the receiver operating characteristic curve	Precision	Recall	Brier loss
Logistic regression	97.8	96.4	98.5^a^	93.4	0.016
Random forest	98.5^b^	97.7	98.1	96.1	0.012^c^
Extra trees	97.9	96.8	98.1	94.2	0.016
Gradient boosting trees	98.4	97.8^d^	97.5	96.5^e^	0.012^c^
Artificial neural network	97.1	95.3	96.6	91.8	0.024

^a^The highest precision.

^b^The highest accuracy.

^c^The least Brier loss.

^d^The highest area under the receiver operating characteristic curve.

^e^The highest recall.

According to Table 4 showing the training times, LR turns out to be superior compared with other models with less than 1-min training time. However, DNN takes up to 30 minutes to train. This could be explained by the complexity level of the 2 algorithms; whereas LR is a very simple and straightforward model based on a linear regression equation, DNN is an architecture that is composed of many neurons, layers, and more complex activation functions.

**Figure 6:**
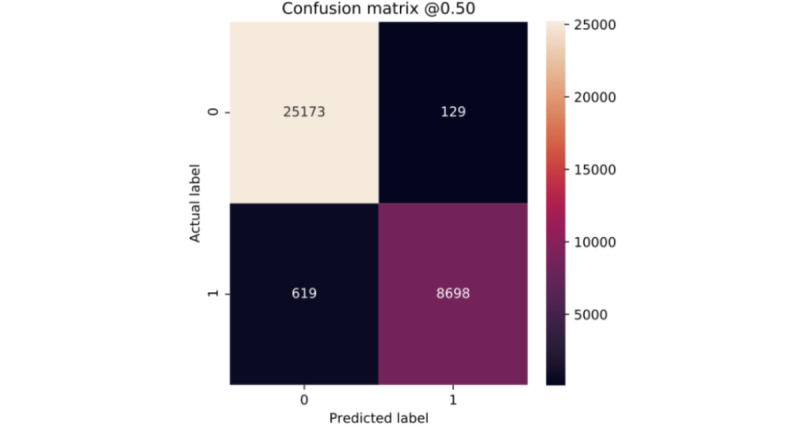
Confusion matrices of logistic regression.

**Figure 7:**
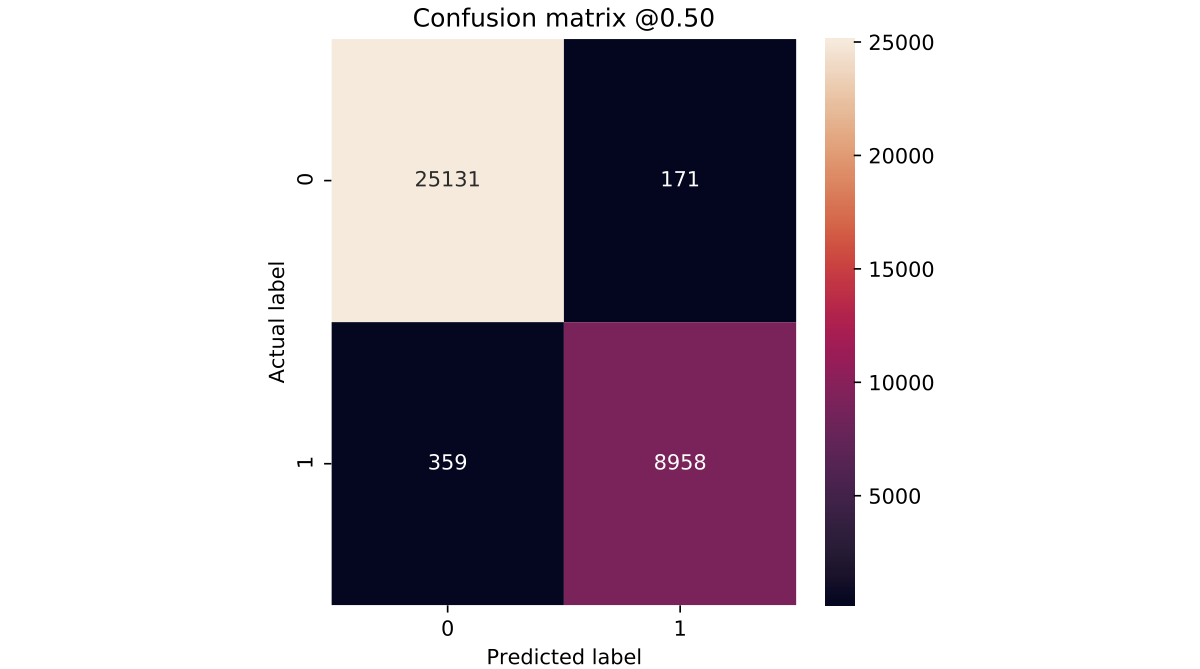
Confusion matrix of random forest.

**Figure 8:**
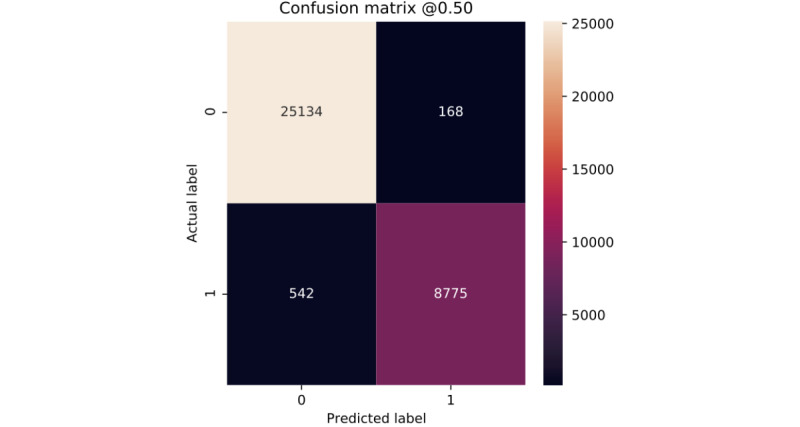
Confusion matrix of extra trees.

**Figure 9:**
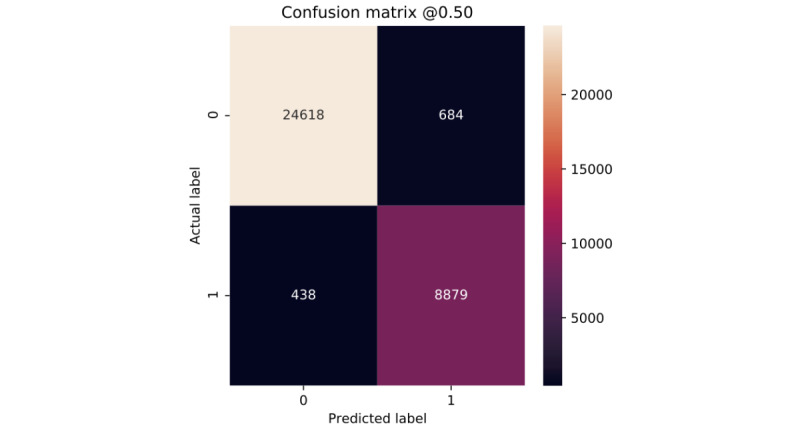
Confusion matrix of gradient boosting trees.

**Figure 10:**
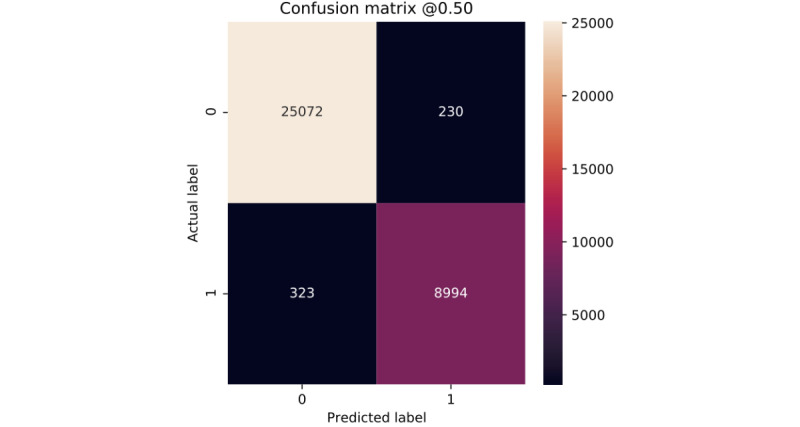
Confusion matrix of artificial neural network.

Clearly, all of our models show very similar behavior for the 2 classes (Figures 6-10). According to the confusion matrices, RF and GBT managed to identify the *deceased* patients with higher accuracy than other algorithms. Compared with other models, DNN classifies a larger number of *deceased* patients as *alive*.

**Table 4:** Training time of machine learning models without Synthetic Minority Oversampling Technique.

Algorithms	Training time (seconds)
Logistic regression	6.6^a^
Random forest	106.8
Extra trees	46.8
Gradient boosting trees	186
Artificial neural network	1277.4

^a^The least training time.

**Figure 11:**
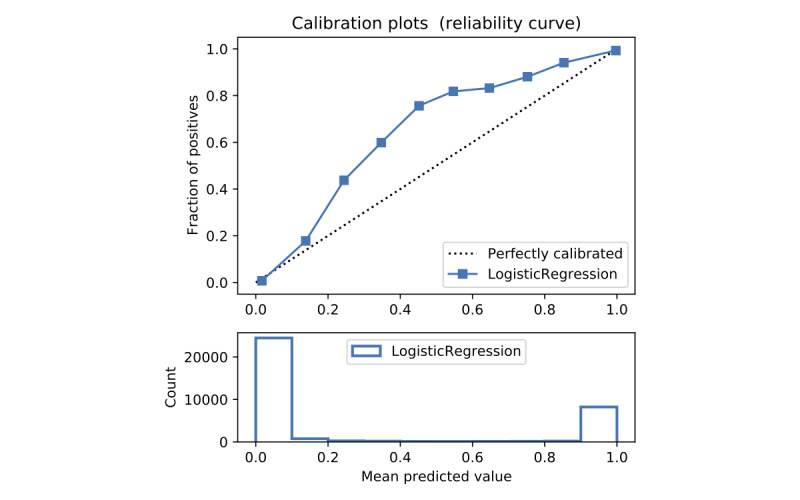
Calibration curve of random forest without Synthetic Minority Oversampling Technique.

**Figure 12:**
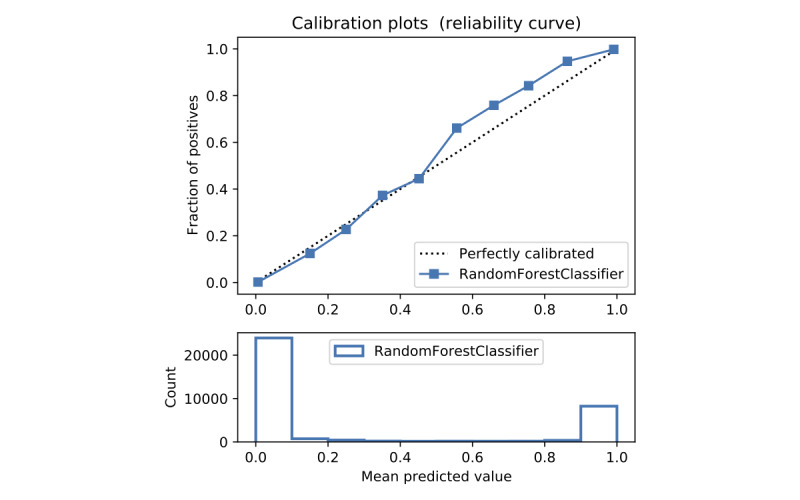
Calibration curve of random forest without Synthetic Minority Oversampling Technique.

**Figure 13:**
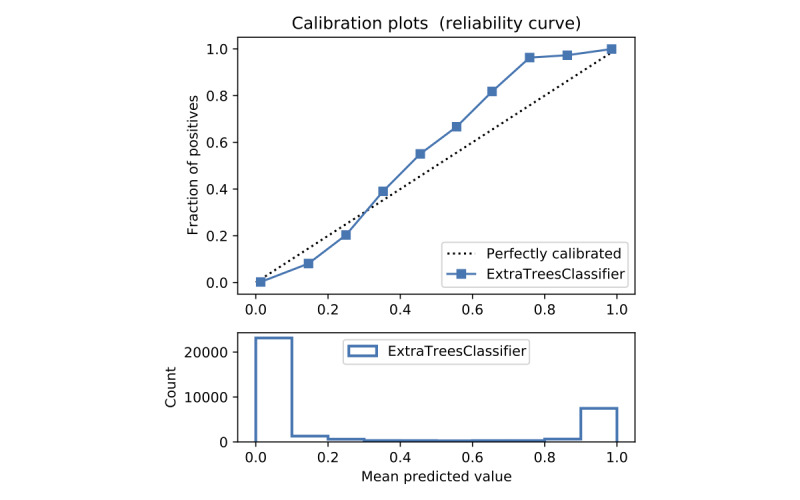
Calibration curve of extra trees without Synthetic Minority Oversampling Technique.

**Figure 14:**
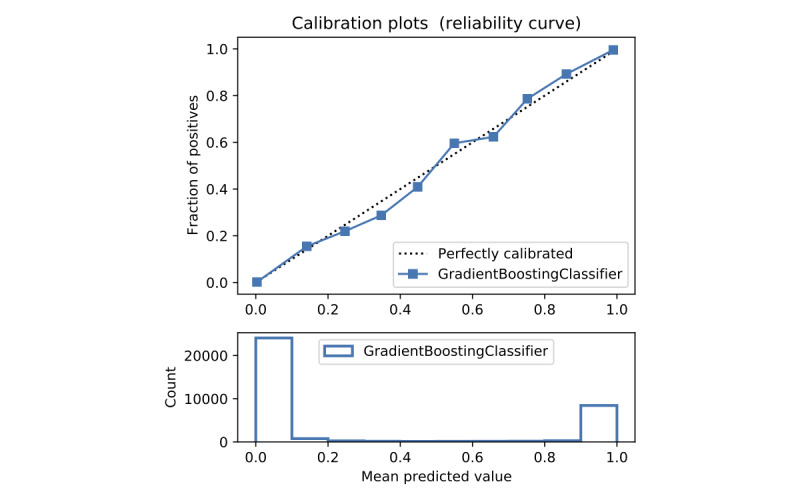
Calibration curve of gradient boosting trees without Synthetic Minority Oversampling Technique.

**Figure 15:**
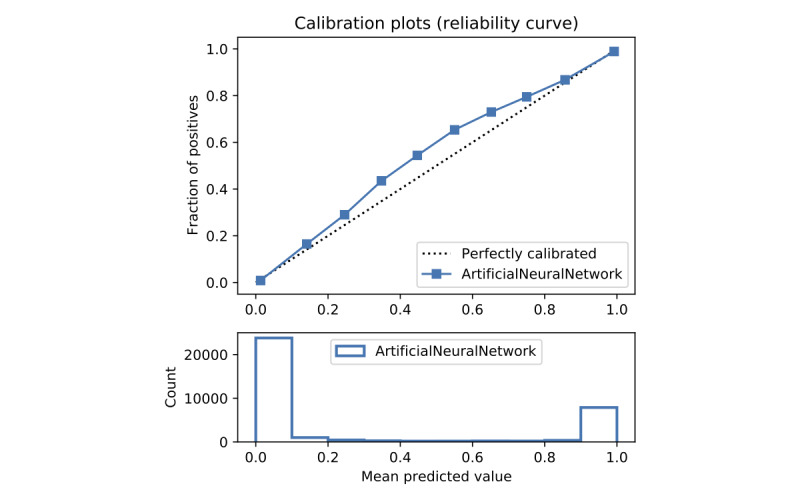
Calibration curve of artificial neural network without Synthetic Minority Oversampling Technique.

**Figure 16:**
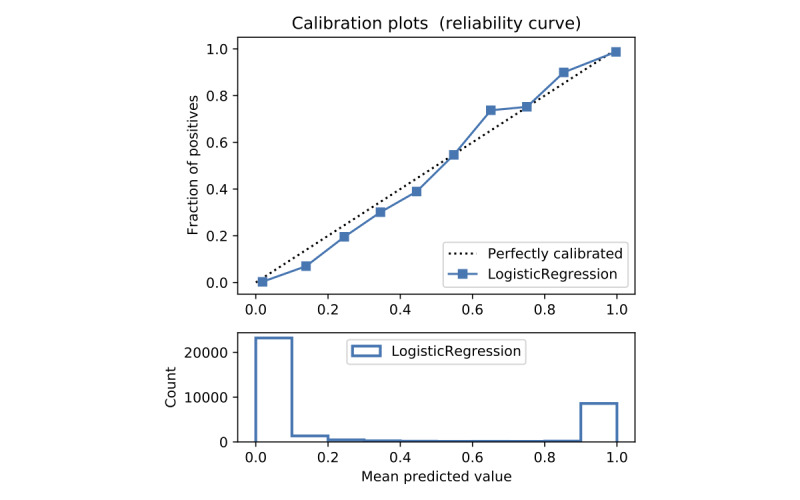
Calibration curve of logistic regression with Synthetic Minority Oversampling Technique.

**Figure 17:**
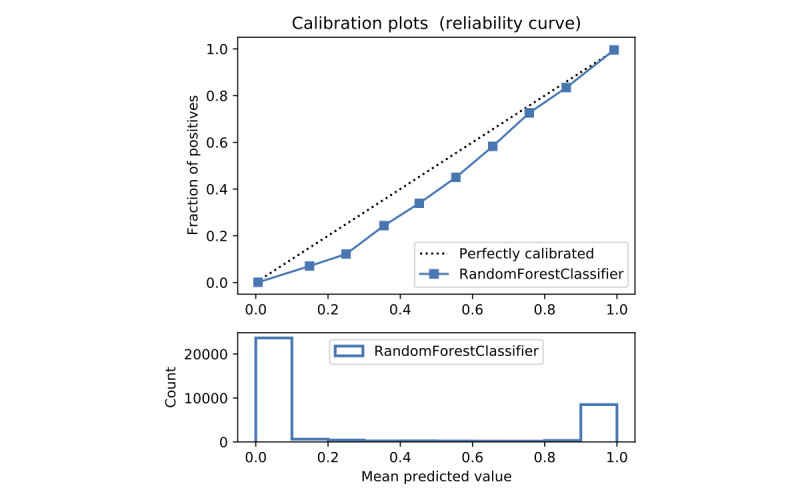
Calibration curve of random forest with Synthetic Minority Oversampling Technique.

**Figure 18:**
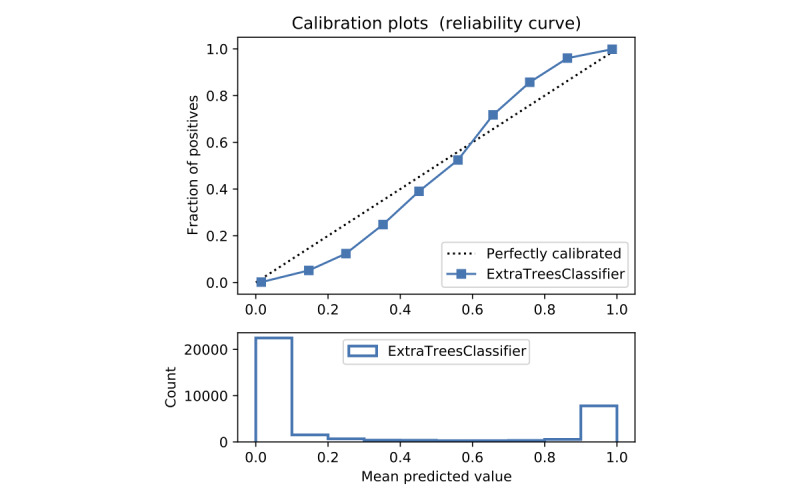
Calibration curve of extra trees with Synthetic Minority Oversampling Technique.

**Figure 19:**
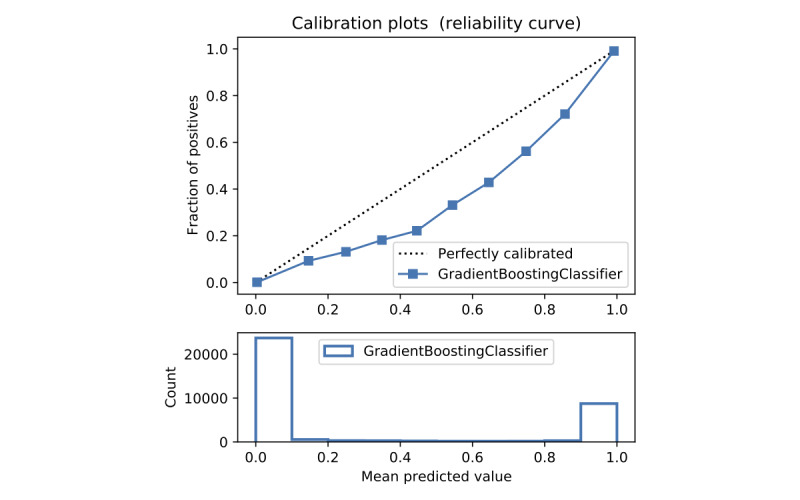
Calibration curve of gradient boosting trees with Synthetic Minority Oversampling Technique.

**Figure 20:**
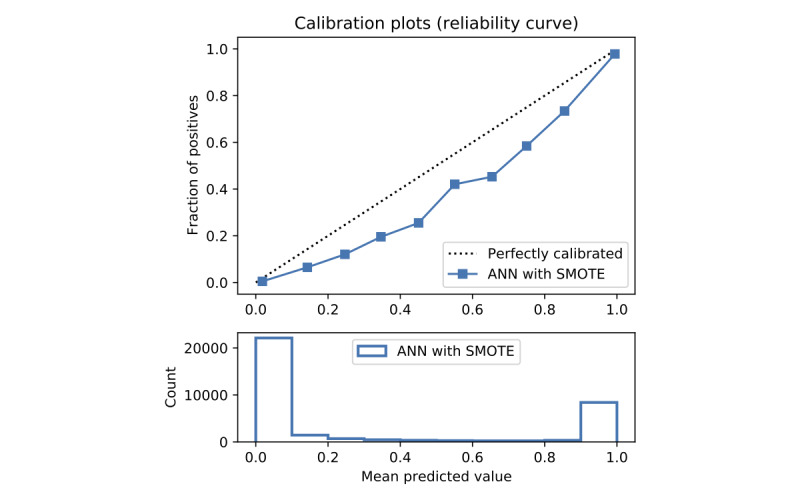
Calibration curve of artificial neural network with Synthetic Minority Oversampling Technique.

In terms of prediction reliability, calibration curves for the 5 models in Figures 11-20 show that LG was the least calibrated compared with the other 4 algorithms, highly overestimating patient death risks at all levels of probabilities. RF was well calibrated for patients with a lower mortality rate and overestimated the risk of death when the probability of risk was more than 50%. ET’s goodness of fit was only seen in the probability of death at 30%, whereas it underestimated and overestimated the risk for patients with lower and higher probabilities of death, respectively. Predictions by GBT and DNN were the most well calibrated, whereas DNN slightly overestimated patients with probabilities of death greater than 10% and less than 90%.

#### Performance With SMOTE

Details of the model performance with SMOTE are presented in Table 5, and their calibration plots are displayed in Figure 8. As can be seen in Table 5, SMOTE slightly improves the performance (in italics) of the 5 models. However, it helps calibrate the predictions of LR significantly. After upsampling, the LR model no longer overestimates death risks of the patient, and its predictions are more closely aligned with the perfectly calibrated line. Meanwhile, ET is now seen as having goodness of fit in predictions of patients with death risk between 50% and 60% but still underestimates and overestimates those with low and high death risks, respectively. On the other hand, RF predictions change from being well calibrated for less than 50% probabilities of death risk and overestimating higher ones into being well calibrated for greater than 80% probabilities of death risk and underestimating the rest. More interestingly, DNN and GBT are subject to adversarial effects from the upsampling technique, generally underestimate the risk.

**Table 5:** Performance metrics of machine learning models with the Synthetic Minority Oversampling Technique.

Algorithms	Accuracy	Area under the receiver operating characteristic curve	Precision	Recall	Brier loss
Logistic regression	98.2	97.4	97.3^a^	95.9	0.015
Random forest	98.4^b^	98.0^c^	96.8	97.3	0.012^d^
Extra trees	98.1	97.4	97.1	95.8	0.016
Gradient boosting trees	98.1	97.9	95.2	97.7^e^	0.014
Artificial neural network	96.7	96.2	93.0	95.1	0.026

^a^The highest precision.

^b^The highest accuracy.

^c^The highest area under the receiver operating characteristic curve.

^d^The least Brier loss.

^e^The highest recall.

In short, SMOTE is only helpful for further improving the model performance and prediction calibration of LG. Meanwhile, using or not using SMOTE does not affect the performance of RF and ET in predicting mortality in patients with CVD. Last, SMOTE introduces an adversarial effect into the GBT and DNN models, making their predictions less reliable, and these 2 models already work well with class imbalanced data.

In terms of training duration, as shown in Table 6, using SMOTE requires more computing time for all the algorithms. However, LR is still the most time-efficient model even when applying SMOTE and produces higher accuracy and better prediction performance in terms of AUROC, recall, and brier loss compared with LR with original data. Furthermore, SMOTE helps LR outperform ET and become the second-best algorithm after RF. Clearly, when introducing SMOTE into the table, ET and LR are worth considering for this data set.

**Table 6:** Training time of machine learning models with the Synthetic Minority Oversampling Technique.

Algorithms	Training time (seconds)
Logistic regression	292.9^a^
Random forest	497.9
Extra trees	347.5
Gradient boosting trees	648.1
Artificial neural network	5480.3

^a^The least training time.

## Discussion

### Principal Findings

This study shows that structured medical and pharmaceutical claims data can be used as input for AI models to accurately predict the mortality risk of individuals with CVD. The LR, RF, ET, GBT, and ANN models trained in this study had high accuracy (ie, 97.0%-98.0%) and discrimination (ie, AUROC 95.0%-98.0%) in predicting the mortality rate, which are much higher than for traditional statistical models such as the Cox Proportional-Hazards model [42] or the models trained with traditional electrical health records [43-45].

Although there was no statistically significant difference in accuracy among the 5 experimental algorithms, the RF model had an advantage over the other models. In addition, the RF model outperformed the other models in terms of recall and brier loss. In terms of discrimination and calibration, the GBT proved to be the most superior. Without SMOTE, LR is unable to make highly calibrated predictions while using SMOTE significantly improves the reliability of the model’s predictions. All models with SMOTE had very high precision (ie, 93.0%-97.0%) and recall (ie, 95.0%-97.0%), particularly when compared with other LR and RF prognostic models that did not deal with class imbalance published in the literature [44,45]. On the other hand, although the ANN had the most moderate performance among the experimental algorithms, it was proven to be efficient even with class imbalanced data. It is also suggested that ANNs are capable of predicting CVD mortality rates more accurately than other ML algorithms if more feature-engineering techniques are applied [46,47], indicating it is a very promising area for further research.

To our knowledge, this is the first study comparing AI algorithms using medical and pharmaceutical claims data to predict mortality in a large general cardiology population. Unlike previously developed ML-based prognostic tools in cardiology that used the clinical information of patients, including clinical features [43-45], our models were trained only on claims data of patients with CVD. These claims data primarily provide information about a patient’s medical scheduling and pharmaceutical dispensing history, which reflect the patient’s disease treatment cost, access patterns, and medications but not the patient’s state of health or other clinical indices. Furthermore, compared with previously published classifiers in cardiology, our models used fewer features and are comparatively more efficient than previously trained models in the general cardiology setting.

### Limitations

Despite high accuracy and strong discrimination, some models, including RF, ET, and ANN, still have not yielded optimal calibrations. This means that the distribution and behavior of the predicted probability is not similar to the distribution and behavior of the probability observed in training data. To increase the reliability of AI algorithms, other techniques should be investigated to better calibrate and improve the performance of these models, especially ANNs.

### Conclusions

We developed, validated, and compared 5 AI architectures to predict the mortality rate of patients with CVD. On the basis of the evaluation results, we can draw the following conclusions or insights that could help with the choice of AI models: (1) without health indices or health condition information, AI architectures are able to accurately predict mortality of patients with CVD before a clinic visit using only medical scheduling and pharmaceutical dispensing claims data; (2) although there was no statistically significant difference in accuracy among the experimental AI algorithms, the tree-based, that is, RF and GBT models have an advantage compared with other models; (3) although the regression-based LR method produces predictions having the least calibration level because of a lack of minority class samples, the upsampling technique, that is, SMOTE helps significantly improve the reliability of this algorithm's predictions; and (iv) tree-based algorithms and densely connected neural networks perform well with class imbalanced data. Finally, this study showed the feasibility and effectiveness of different AI architectures based on structured medical scheduling and pharmaceutical dispensing claims data in identifying patients with CVD who had a risk of mortality; AI algorithms can be a useful tool for precise decision making. Future research, considering the promising potential of the ANN, should focus on improving the prediction performance of this algorithm. It is suggested that ANNs are capable of predicting CVD mortality rates more accurately than other ML algorithms if more feature-engineering techniques are applied, indicating they are a very promising area for further research.

## Abbreviations

ANN: artificial neural network
AUROC: area under the receiver operating characteristic curve
CVD: cardiovascular disease
EHR: electronic health record
ET: extra trees
GBT: gradient boosting trees
LR: logistic regression
MBS: Medicare Benefits Schedule
ML: machine learning
PBS: Pharmaceutical Benefits Scheme
RF: random forest
SMOTE: Synthetic Minority Oversampling Technique
